# Evaluation of the cytotoxicity, mutagenicity and antimutagenicity of a natural antidepressant, *Hypericum perforatum* L. (St. John’s wort), on vegetal and animal test systems

**DOI:** 10.1186/1472-6882-13-97

**Published:** 2013-05-06

**Authors:** Ana Paula Peron, Rosinete Gonçalves Mariucci, Igor Vivian de Almeida, Elisângela Düsman, Mário Sérgio Mantovani, Veronica Elisa Pimenta Vicentini

**Affiliations:** 1 Department of Biotechnology, Genetics and Cell Biology, State University of Maringá, Avenida Colombo 5790, Bloco H67 (11), Jardim Universitário, Maringá, Paraná CEP: 87020-900, Brazil; 2 Department of Biotechnology, Genetics and Cell Biology, State University of Maringá, Maringá, Paraná, Brazil; 3 Department of Biotechnology, Genetics and Cell Biology, State University of Maringá, Maringá, Paraná, Brazil; 4 Department of Biotechnology, Genetics and Cell Biology, State University of Maringá, Maringá, Paraná, Brazil; 5 Department of General Biology, State University of Londrina, Londrina, Paraná, Brazil; 6 Department of Biotechnology, Genetics and Cell Biology, State University of Maringá, Maringá, Paraná, Brazil

**Keywords:** Chromosomal aberration, Pharmacological action, Wistar rats, Medicinal plant, *Allium cepa* L., Mitotic index

## Abstract

**Background:**

St. John’s wort (*Hypericum perforatum* L.) is an herbaceous plant that is native to Europe, West Asia and North Africa and that is recognized and used worldwide for the treatment of mild and moderate depression. It also has been shown to be therapeutic for the treatment of burns, bruises and swelling and can be used for its wound healing, antiviral, antimicrobial, antioxidant, analgesic, hepato-protective and anxiolytic properties. The aim of this study was to evaluate the potential cytotoxic, mutagenic and antimutagenic action of *H. Perforatum*.

**Methods:**

Meristematic cells were used as the test system for *Allium cepa* L., and bone marrow cells from *Rattus norvegicus*, *ex vivo*, were used to calculate the mitotic index and the percentage of chromosomal aberration. Statistical analysis was performed using the chi-square test.

**Results:**

This medicinal plant had no cytotoxic potential in the vegetal test system evaluated. In the animal test system, none of the acute treatments, including intraperitoneal gavage and subchronic gavage, were cytotoxic or mutagenic. Moreover, this plant presented antimutagenic activity against the clastogenic action of cyclophosphamide, as confirmed in pre-treatment (76% reduction in damage), simultaneous treatment (95%) and post-treatment (97%).

**Conclusions:**

Thus, the results of this study suggest that the administration of *H. perforatum*, especially by gavage similar to oral consumption used by humans, is safe and with beneficial antimutagenic potential.

## Background

*Hypericum perforatum* L., popularly called St. John’s wort (SJW), is a perennial herbaceous plant that has yellow flowers and is native to Europe, West Asia and North Africa, but was also naturalized in the Americas and Australia. Its name refers to the period of flowering and harvest, which takes place around St. John’s Day, June 24. The genus *Hypericum* belongs to the family Clusiaceae and contains approximately 370 species [1]. In order for the flowers and aerial parts to be suitably used, they should both be immediately dried in a drying chamber after harvesting to avoid degradation of their active components [2].

Because of its broad action on the nervous system and the positive impact on the treatment of mild to moderate depression [1-4], consumption of this plant has increased dramatically in recent years in several countries. Even with the difficulty of estimating these numbers, Linde [1] showed that more than 9.5 million packages of SJW capsules were sold in Europe between April 2007 and March 2008. The main markets for SJW are Germany (3.8 million), Russia (2.2 million) and Poland (1.6 million), which together represent 79% of sales in Europe. These numbers had been higher before the imposition of stricter standards for prescribing this medicinal plant. In the United States in 2007, the market for dietary supplements derived from SJW reached approximately 8.1 million dollars.

Many species of the genus *Hypericum* have medicinal properties and were previously used in ancient Greece for burns and leg pain and also for their antidiuretic and antimalarial properties. *Hypericum* species were also applied to external areas and were recommended to be consumed with wine to combat poisonous reptiles [5].

Currently, several species of the genus are being used as a medicine based on knowledge gained from investigations in several countries. Thus, this genus has healing and diuretic properties and is used to treat kidney, urinary bladder, liver, and migraine issues. In addition, it is anti-tussive and anthelmintic. This genus also has sedative and calming effects and acts as an antidepressant [6-8].

*H. perforatum*, a representative species of the genus, has been included in traditional medicines in various countries [9]. Decoctions of floral parts and *H. perforatum* have been used for the treatment of disorders of the digestive tract, urinary tract, respiratory system, heart, liver, joints and mental health [10-18]. Maury et al. [19] identified 3-hydroxy lauric acid, which is found in field grown *H. perforatum,* as having anti-HIV activity.

The genus *Hypericum* has an exceptionally diverse and complex chemical composition, and *H. perforatum* has been found to contain several classes of components similar to most plants, including essential oils, flavonoids, polyphenols, procyanidins, tannins, fenilpropanos, xanthones and porphyrins [20]. In addition to these components, two classes of very active constituents were found exclusively in this genus, floroglucinoides, which are mainly hyperforin and adhiperforin, and naftodiantrones, such as hypericin and pseudohipericin [21].

Hypericins and hiperforins are primarily responsible for the antidepressant activity of SJW because they inhibit the transporters that recapture norepinephrine, serotonin and dopamine that are released into the synaptic cleft of neurons [22,23]. In methanolic or ethanolic extracts of SJW, the concentration of hyperforin and adhiperforin can reach 6%, but these concentrations vary widely depending on the concentration of the alcohol [1].

The flavonoid glycosides comprise the second largest group of active metabolites in SJW and are mainly represented by rutin, hyperoside, isoquercetin and quercitrin. These metabolites have some antidepressant properties, but most often act as co-effectors, increasing the pharmacological properties of other substances, such as hypericins [24].

There use of medicinal plants by the world’s population is growing. Because of the consumption of teas or extracts of SJW for the treatment of mild and moderate depression, or as a co-adjuvant with conventional drugs, it is of interest to determine whether the use of this plant has any benefit or harm at the cytological and chromosomal levels. Studies involving the antimutagenic potential of medicinal plants are important because these plants can be used as a means of treatment without interfering in a normal lifestyle or result in the intake of an excessive number of synthetic substances. The aim of this study was to evaluate the potential cytotoxic, mutagenic and antimutagenic/protector functions of *H. perforatum*. As a test system, we used meristematic cells from *Allium cepa* L. and bone marrow cells from *Rattus norvegicus*, *in vivo*. Thus, this study stands out from previously conducted studies because of its investigation of the antimutagenic activities of SJW against cyclophosamide, a mutagenic chemotherapeutic agent.

## Methods

### Treatment solutions

The *Hypericum perforatum* L. (SJW) was extracted from capsules (300 mg) (dried leaves and ground) added to an excipient starch (Herbarium Lab), equivalent to 0.9 mg (0.3%) of hipericin, and was dissolved in water at three concentrations: 0.3, 3.0 and 30.0 mg/mL. Concentrations were chosen based on an extrapolation of a daily dose consumed by humans.

The cytostatic drug cyclophosphamide was diluted with water at a concentration of 1.5 mg/mL and was used as a positive control.

### *Allium cepa* L*.* root-tip cells

The experiments were conducted using the Feulgen reaction and Schiff’s reagent for staining, according to the methodology originally introduced by Levan in 1949 (in Fiskesjö [25]).

The onion bulbs were placed for rooting in bottles with water at room temperature and aerated in the dark. Before each treatment, three roots were collected and fixed (3 methanol: 1 acetic acid) to serve as control bulbs (Co). The remaining roots were then placed in treatment solutions with three concentrations of SJW 0.3, 3.0 and 30.0 mg/mL) for 24 hours. After the treatment period, three roots were withdrawn from each onion and fixed (Tr). The remaining roots were washed, and the bulbs were again placed in water for 24 hours to recover from any damage; then, the remaining roots were removed and fixed (Re). The negative control onions remained in filtered water throughout the sampling time (CO^-^).

The slides were analyzed by light microscopy (40x objective) in “blind tests”. To determine the mitotic index (MI-%), five bulbs were used in each control and treatment group. One thousand cells per bulb were analyzed, totaling 5,000 cells for the control, treatment and recovery groups. Statistical analysis was performed using the chi-square test (n = 5, α = 0.05).

### Wistar rat bone marrow cells

Six Wistar rats (*Rattus norvegicus*), three males and three females for each group, were obtained from the Central Vivarium of the State University of Maringá (UEM). Experiments were carried out using 35-day old rats weighing approximately 100 g (bw).

### Ethics statement

During the experimentation period, the animals remained under controlled temperatures of approximately 25°C, with humidity at approximately 50% and with a photoperiod of 12 hours light/dark. Furthermore, all Ethical Principles, Protocols and Regulations on Experimentation with Laboratory Animals were used according to the standards established internationally and by the approved project by the Institutional Ethics Committee of State University of Maringá (UEM), the Ethics Committee on Animal Use in Experimentation (CEAE)/UEM, following the Ethical Principles for Animal Experimentation established by the Brazilian College of Animal Experimentation (COBEA), as well as the specific treatment and collections protocols made to chromosomal aberration test.

### Acute treatment (intraperitoneal and gavage)

For mutagenesis analysis, rats were treated with an intraperitoneal injection (0.3, 3.0 and 30.0 mg SJW/mL) or with via gavage (3.0 and 30.0 mg SJW/mL), with 1 mL of the water or treatment solutions/100 g bw. The animals were euthanized 24 hours after treatments.

For antimutagenic analysis, the SJW treatment was administered by gavage at a concentration of 30.0 mg/mL prior to (pre-treatment), concurrent with (simultaneous treatment) or after (post-treatment) the application of an intraperitoneal injection of cyclophosphamide 1.5 mg/mL. The high concentration of SJW was used in the antimutagenicity tests because, in general, this treatment has presented a lower percentage of chromosomal abnormalities and because the public’s increasing exposure to herbal medicines warrants testing greater amounts.

### Subchronic treatment (gavage)

Wistar rats were submitted to a subchronic treatment for seven days. Control group received 1 mL water daily via gavage, and the treatment groups received the same quantity of SJW treatment solutions at concentrations of 0.3, 3.0 and 30.0 mg/mL.

The rats were kept in their cages, and food and water were changed daily at the same time. On the eighth day, the rats were euthanized.

### Chromosomal aberration test

A chromosomal aberration test was performed on the bone marrow cells from Wistar rats using the Ford and Hamerton method [26], with some modifications. The mitotic cells were interrupted in metaphase with the intraperitoneal administration of 0.5 mL/100 g bw of colchicine (0.16%), half an hour before euthanasia.

The analysis of the slides was performed by a light microscope, analyzing 100 cells in metaphase per animal, totaling 600 cells each for the control and treatment groups. Cells were assessed for the appearance of alterations, such as gaps, breaks, fragments, and others. The mitotic index (MI) for the cytotoxicity evaluation was calculated from 5,000 cells from each sex, totaling 10,000 cells per group. The MI was calculated as a percentage as follows: the number of dividing cells divided by the total number of cells present in the fields. The statistical calculation was performed using the chi-square test (n = 6, α = 0.05).

## Results

### *Allium cepa* L*.* root-tip cells

**Figure 1:**
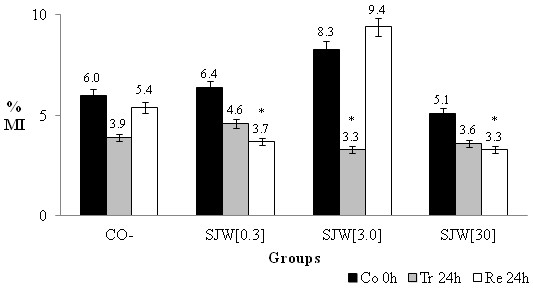
**The mean percentage and standard deviation of the mitotic index (MI) for the negative control group (CO-) and the group treated with St. John´s Wort (SJW) at three concentrations (mg/mL).** Treatment time: Control (Co) = 0 h, Treated (Tr) = 24 h, Recovery (Re) = 24 h. * The results are statistically significant in relation to Recovery (24 h) SJW [3.0].

Figure 1 presents the results of the mitotic index and shows that treatments with different SJW concentrations at various sampling times (Co 0 h, Tr 24 h and Re 24 h) were similar to the negative control. However, we observed a significant increase (*χ*2 = 11.27) in the number of dividing cells during the recovery of the group treated with the 3.0 mg/mL concentration of SJW, from 3.3% (Tr 24 h) to 9.4% (Re 24 h). There were also significantly higher rates of recoveries at concentrations of 0.3 mg/mL (*χ*2 = 8.78) and 30.0 mg/mL (*χ*2 = 3.96).

### Wistar rat bone marrow cells

**Figure 2:**
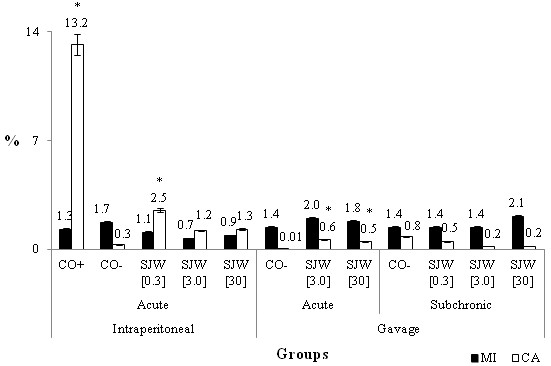
**The mean percentage and standard deviation of the mitotic index (MI) and chromosomal alteration (CA) of acute and subchronic gavage and intraperitoneal treatments for the negative (CO-) and positive (CO+) control groups and for the group treated with three concentrations of St. John´s wort (SJW) (mg/mL).** * The results are statistically significant in relation to CO-.

**Figure 3:**
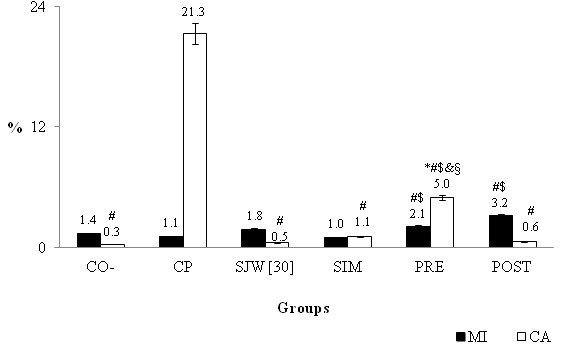
**The mean percentage and standard deviation of the mitotic index (MI) and chromosomal alteration (CA) for the negative (CO-) control group and the group treated with St. John´s wort [30.0 mg/mL] simultaneously (SIM), previously (PRE) and after (POST) the application of cyclophosphamide (CP) [1.5 mg/mL].** * The results are statistically significant in relation to CO-. # The results statistically significant in relation to CP. $ The results are statistically significant in relation to Simultaneous Treatment. & The results are statistically significant in relation to Post-treatment. § The results are statistically significant in relation to SJW [30].

For all tests with bone marrow cells from Wistar rats, no differences in the mitotic index were observed between the control groups and the treatments with SJW (Figure 2), demonstrating the absence of cytotoxic activity of *H. perforatum*. Only in the antimutagenicity test (Figure 3) did we observe a statistically higher mitotic index in the post-treatment group when compared with the simultaneous treatments (*χ*2 = 4.48) and cyclophosphamide (*χ*2 = 4.69).

In the acute test for mutagenicity via gavage (Figure 2), we observed a significantly higher number of chromosomal aberrations at the concentrations of 3.0 mg/mL (*χ*2 = 34.81) and 30.0 mg/mL (*χ*2 = 24.01) of SJW, compared to the negative control. In turn, the acute test for mutagenicity of intraperitoneally injected SJW (Figure 2) showed that the lowest concentration evaluated (0.3 mg/mL) induced a statistically significant increase in the number of chromosomal aberrations over the negative control (*χ*2 = 16.13). Mutagenicity was tested after subchronic gavage (Figure 2), and the three concentrations of SJW evaluated did not induce an increase in the percentage of chromosomal aberrations compared to the negative control. All concentrations of SJW in all treatments evaluated were different than the positive control.

In the antimutagenicity test (Figure 3), simultaneous treatment and post-treatment reduced the percentage of damage induced by cyclophosphamide by a significant amount (SIM - *χ*2 = 370.94; POST - *χ*2 = 714.15) when compared to the level observed for the negative control and for the SJW treatment alone. The pre-treatment also significantly reduced the percentage of drug-induced damage (PRE - *χ*2 = 3.13), but did not reached the values observed in the negative control (*χ*2 = 53.13), which was different from the treatment with SJW alone (*χ*2 = 40.50) and also different from the simultaneous treatments (*χ*2 = 13.83) and post-treatment (*χ*2 = 3.87).

Note that the response between the sexes in the treated animals in these experiments was similar regarding mitotic index and percentage of chromosomal abnormalities.

## Discussion

Chromosomal aberrations have been recognized for a long time as important biomarkers for human exposure to genotoxic chemical agents and ionizing radiation. Structural and numerical aberrations have been associated with poor health; for example, congenital abnormalities in newborns and neoplasias in humans. The genetic instability, or chromosomal instability, also seems to be related to cancer, although the mechanisms involved in this process are not yet fully elucidated.

The interest in organic extracts of medicinal plants or parts of the plants, such as SJW, has increased because these extracts are relatively safe to use, show a good acceptance among consumers and can possibly be used for many different functions [5].

The results of this study indicate that SJW has no cytotoxic effect on the root meristematic cells of *A. cepa* and no effect on bone marrow cells from Wistar rats. In the vegetal system (Figure 1), we observed that at all concentrations tested, *H. perforatum* caused no significant decrease in cell division after 24 hours of treatment. However, after the recovery treatment with a concentration of 3.0 mg/mL of SJW, there was increased cellular proliferation, which was statistically significant with respect to treatment with SJW alone and the recoveries of the other treatments. This finding indicates that despite the inhibition of cell division to less than half compared to control (8.3 to 3.3), the cells recovered to a division index greater (9.4) than that obtained for the control (8.3).

Other studies have also shown that SJW or its components can reduce cell divisions. Schempp et al. [27] determined that hyperforin, a major constituent of SJW, inhibits the proliferation of epithelial cells and human peripheral blood mononuclear cells, even when stimulated by phytohemagglutinin. Hyperforin also inhibited the growth of autologous breast cancer cells, MT-450, in Wistar rats *in vivo*[28]. In this respect, the inhibitory effect of cell proliferation in cancer cells is a major benefit of this medicinal plant. Alecu et al. [29], Patocka [30], Kusari et al. [31] and Xu and Leung [32] also demonstrated that hypericin, another important component of SJW, inhibited the growth of glioma cells *in vitro* and was also capable of inducing cell death through the inhibition of protein kinase C (PKC), which regulates the formation and growth of tumors.

The anti-proliferative action of crude methanol extracts of *H. caprifoliatum*, *H. carinatum*, *H. connatum*, *H. myrianthum*, *H. polyanthemum* and *H. ternum* have been confirmed in two cell lines, cells of human colon carcinoma and lung carcinoma [33]. Other anti-proliferative activities of the methanol extract of *H. perforatum* have been observed in leukemic cells (K562 and U937), human colon carcinoma (HT-29) and cell lung carcinoma (H-460) [33,34]. Compounds isolated from *H. chinense* have demonstrated enhanced cytotoxicity against human cancer cell lines, including multidrug resistant cells [35,36]. Otogirone and erectquione B chloroform extracts from whole plants of *H. erectum* have been shown to inhibit the growth of chloroquine-sensitive strains of *Plasmodium falciparum*[37].

In the Wistar rat test system (Figure 2), after acute or subchronic gavage, SWJ was not cytotoxic, but there was no significant increase of cell division. After intraperitoneal treatment, there was no statistically significant decrease in the rate of cell division, similar to what happened in the plant test system. There was no cytotoxic activity of *H. perforatum* in mammals. Similar to the data in the present study, and the results presented in the revised work of Hammerness et al. [38], SJW showed no toxic effects at doses up to 5,000 mg/kg in mice and rats. Additionally, in rats and dogs, there were only non-specific symptoms, such as weight loss, in long-term studies.

Other studies have also shown no harmful action of SWJ in subchronic treatment. Rayburn et al. [39] showed that prenatal exposure to a therapeutic dose of SJW caused no deficit in long-term behavioral tasks in the offspring of mice. Rayburn et al. [40] also indicated that maternal administration of SJW before and during pregnancy did not affect the growth or physical maturation of the progeny of exposed rats.

Evaluation of the mutagenic activity of SJW in this study showed that this plant had no mutagenic potential in subchronic gavage treatments (0.3, 3.0 and 30.0 mg/mL) (Figure 2). The acute gavage treatments (3.0 and 30.0 mg/mL) with SJW were mutagenic, as evidenced by the fact that their mutagenicity was significantly different from the negative control. Although the percentage of chromosomal alteration due to these treatments was very low ([3.0] = 0.6% and [30] = 0.5%), and the percentage of abnormalities compared to the negative control was practically nil (0.01%).

In a previous study, an aqueous ethanolic extract of *Hypericum* also showed no genotoxicity *in vitro*, using Chinese hamster embryo cells and hypoxanthine guanidine phosphoribosyl transferase, DNA synthesis and cell transformation assays, or *in vivo*, by spot tests in mice and chromosome aberration assays [41]. Ndhlala et al. [42] determined that *H. aethiopicum* was not mutagenic by the Ames test with *Salmonella typhimurium,* and they found that it had antifungal activity against *Candida albicans*.

These data also corroborate the work of Miadokova et al. [43]. Using different test systems to evaluate the effect of hypericin, an isolated compound of SJW, they also found no genotoxicity or antigenotoxicity activities for this substance in the Ames test. When *Saccharomyces cerevisiae* was used for testing, no increase in the number of mitotic crossovers or point mutations was observed, and there was no difference in the frequency of structural chromosome aberrations in three cell lines (HepG2, V79 and VH10).

The negative results for the mutagenicity of subchronic treatments of SJW agree with the lack of effect on fertility, reproduction, teratogenesis and mutagenesis with doses of up to 300 mg *Hypericum*/kg in rats and dogs, which were presented by Alonso [44]. This finding was similar to those using a 30.0 mg dose of SJW/100 g weight in Wistar rats in the present study.

Regarding the evaluation of mutagenic activity of intraperitoneally injected SJW (Figure 2), the results of this study indicate that the concentrations of 3.0 and 30.0 mg/mL were not mutagenic. It is noteworthy that the lowest concentration of SJW (0.3 mg/mL) showed a percentage of chromosomal abnormalities that was significantly different from the negative control (CO- = 0.3% and [0.3] = 2.5%). Nevertheless, this percentage of chromosomal abnormalities was low (2.5%) considering that the percentage of basal changes in rats is 2%.

However, this percentage of aberrations (2.5%) in the intraperitoneal test ([0.3]) was the highest among all tests with SJW, either by gavage or intraperitoneal injection. In this regard, it can be noted that intraperitoneal administration has a rapid absorption rate due to the large absorptive surface of the abdominal cavity [45]. Thus, the SJW quickly runs through the bloodstream and acts on the bone marrow, resulting in greater damage to DNA. Moreover, this fact can be confirmed by comparing the mitotic index of SJW treatment with intraperitoneal administration (IM [0.3] = 1.15, [3.0] = 0.67, [30] = 0.91) to treatment by gavage; the cells were more affected by injection of SJW rather than administration by gavage (acute MI [3.0] = 2.0 and [30] = 1.78; subchronic IM [0.3] = 1.4, [3.0] = 1.4 and [30] = 2.1). In the administration by gavage, similar to oral ingestion in people, the compound enters the portal circulation and passes through the liver, where it can be metabolized [45] and may have its cytotoxicity/mutagenicity decreased, and then affects the bone marrow. Thus, the higher number of chromosomal alterations observed with treatment with an intraperitoneal injection of *H. perforatum* may be due to the presence of secondary metabolites, such as quercetin, which may be responsible for the genotoxic activity of SJW in *Salmonella typhimurium*[38,46].

The anticancer properties of SJW have become the focus of many studies because many cancer patients were, are using extracts from this plant for the treatment of depressive symptoms developed with the discovery of disease [21]. Thus, the study of the antimutagenic activity of this substance is as important as the study of the anti-proliferative effect; however, the scientific literature does not contain many studies describing the antimutagenic properties of SJW or its components.

In an antimutagenicity test (Figure 3), the results showed that SJW was antimutagenic, reducing the percentages of chromosomal alteration due to cyclophosphamide (21.3%) by 76% in pre-treatment (5.0%), 95% in simultaneous treatment (1.1%) and 97% by post-treatment (0.6%). The negative control was statistically similar to SJW treatment alone, post-treatment and simultaneous treatment. The potential antimutagenic effects (>75%) of dianthrone rich acetone and aqueous methanol extracts of the aerial flowering parts of *H. perforatum* have also been tested against 2-aminofluorene, similar to the present study, by the Ames *Salmonella* histidine reversion assay, with TA98 and TA100 strains of *Salmonella typhimurium*[47]. Similar results were also obtained using the green alga *Chlamydomonas reinhardtii*, which was not mutagenic and reduced toxicity and mutagenicity induced by methyl methane sulfonate [43].

Bhatia et al. [47] suggested that *H. perforatum*, because of its antimutagenic property, can be beneficial for the prevention of cancer. Schwarz et al. [48] also found that a commercial preparation of an extract from *H. perforatum,* the isoform CYP1A1, showed anticancer potential by serving as a potent inhibitor of a key enzyme activator of procarcinogens in humans.

The antimutagenic activity of SJW may be due to the combined action of its components, especially hypericin, hyperforin and phenolic compounds. As indicated by Hostanska et al. [49], in tests of anticarcinogenic properties, many act in a synergistic manner (e.g., hypericin and hyperforin) by exerting anti-proliferative activity. Many components also have cytostatic and apoptotic activities (phenolic compounds) on cells whose genetic material has been severely compromised by the clastogenic action of cyclophosphamide, as indicated by a smaller number of chromosomal damages observed in the metaphases analyzed. According to Theodossiou et al. [50], hypericin is phototoxic and has an intricate mechanism involving key proteins, vital enzymes, organelle membranes and changes in cellular homeostasis that can lead to cell death, which occurs mainly by the induction of apoptosis and/or necrosis.

The post-treatment mechanism of action occurs when the antimutagenic substance acts upon the process that induces the formation of mutations or the process that repairs DNA damage [51-53].

Furthermore, Bhatia et al. [47] also justified the antimutagenic activity of extracts of *H. perforatum* by the presence of phenolic compounds in the plant. These compounds, and, consequently, the high antioxidant activity of these extracts, can interfere with the metabolic activation of pro-mutagens and can act as a blocking agent, forming adducts with final mutagens. *H. perforatum* can also interact with the active groups of mutagens, can protect the sites in DNA that would be affected by the agent, or promote the capture of free radicals [54]. This mechanism, called desmutagen, can be simultaneously tested. Thus, in the present study, the antimutagenic activity of *H. perforatum* (95%) on cells that have been treated with cyclophosphamide simultaneously may be explained by the fact that SJW acts directly on the compounds that induce mutations in DNA, inactivating them chemically or enzymatically, and may inhibit the metabolic activation of pro-mutagenic or may scavenge reactive molecules, as explained by Kada et al. [51] and Kuroda et al. [53].

The protective activity of SJW was also confirmed by Jang et al. [55], using an inducer of cellular damage, hydrogen peroxide (H_2_O_2_). Human neuroblastoma cells pre-treated with SJW prior to H_2_O_2_ exposure showed a decreased occurrence of apoptotic features and inhibited an H_2_O_2_-induced increase in caspase-3 enzyme activity. Moreover, the pretreatment of PC12 cells with a flavonoid-rich extract of *H. perforatum* (FEHP) prior to H_2_O_2_ exposure elevated the cell viability, decreased the levels of lactate dehydrogenase release and decreased the occurrence of apoptotic cells. Additionally, the intensity of H_2_O_2_-induced DNA laddering was inhibited in a dose-dependent fashion by a DNA fragmentation assay [56]. These results suggested that FEHP might be useful in the treatment of oxidative stress-related neurodegenerative diseases, such as Parkinson’s disease and Alzheimer’s disease.

Furthermore, An et al. [57] also showed that 1,3,5,6-tetrahydroxyxanthone and I3, II8-biapigenin, isolated from the methanolic extract of the whole plant of *H. erectum*, had moderate hepatoprotective activity against tacrine-induced cytotoxicity in HepG2 cells.

## Conclusions

The results of this study indicate that *H. perforatum* is not cytotoxic or mutagenic at the times, concentrations and types of treatments studied. Thus, it does not appear to be harmful to the cells of organisms that have been exposed to it. Furthermore, this plant showed antimutagenic properties against the chemotherapeutic drug cyclophosphamide, suggesting that in addition to the medicinal properties shown in several other studies, it also has a protective effect on the DNA within the bone marrow cells of treated rats. Future studies should be conducted to investigate whether SJW interferes with the effectiveness of cyclophosphamide chemotherapy. Thus, *H. perforatum* could be indicated as an antidepressant and could be protective for those individuals undergoing treatment with cyclophosphamide.

## Competing interests

The authors declare that there are no conflicts of interest.

## Authors’ contributions

APP and RGM conducted tests with *Allium cepa* L. and Wistar rats and examined the slides. IVA and ED participated in the design of the study, performed the statistical analysis and wrote the manuscript. MSM and VEPV conceived of the study, participated in its design and coordination and helped to draft the manuscript. All authors read and approved the final manuscript.

## Pre-publication history

The pre-publication history for this paper can be accessed here:

http://www.biomedcentral.com/1472-6882/13/97/prepub
